# Environmental Nanoparticles Reach Human Fetal Brains

**DOI:** 10.3390/biomedicines10020410

**Published:** 2022-02-09

**Authors:** Lilian Calderón-Garcidueñas, Ángel Augusto Pérez-Calatayud, Angélica González-Maciel, Rafael Reynoso-Robles, Héctor G. Silva-Pereyra, Andrea Ramos-Morales, Ricardo Torres-Jardón, Candelario de Jesús Soberanes-Cerino, Raúl Carrillo-Esper, Jesús Carlos Briones-Garduño, Yazmín del Socorro Conde-Gutiérrez

**Affiliations:** 1 Department of Biomedical & Pharmaceutical Sciences, College of Health, The University of Montana, Missoula, MT 59812, USA; 2 Universidad del Valle de México, Mexico City 14370, Mexico; 3 División de Áreas Críticas, Hospital General de Mexico Dr. Eduardo Liceaga, Mexico City 06720, Mexico; gmemiinv@gmail.com (Á.A.P.-C.); drcarlosbriones@yahoo.com.mx (J.C.B.-G.); 4 Instituto Nacional de Pediatría, Mexico City 04530, Mexico; agonzalezmaciel@yahoo.com (A.G.-M.); reynosoraf@yahoo.com (R.R.-R.); destrellaramos@yahoo.com.mx (A.R.-M.); 5 Instituto Potosino de Investigación Científica Y Tecnológica A. C., San Luis Potosi 78216, Mexico; hector.silva@ipicyt.edu.mx; 6 Instituto de Ciencias de la Atmósfera y Cambio Climático, Universidad Nacional Autónoma de Mexico, Mexico City 04510, Mexico; rtorres@unam.mx; 7 Residente de Cuarto año de Ginecología y Obstetricia, Hospital Regional de Alta Especialidad Dr. Gustavo A. Rovirosa Pérez, Villahermosa 86020, Mexico; cjesobc@live.com.mx (C.d.J.S.-C.); yamicondesa@hotmail.com (Y.d.S.C.-G.); 8 Centro Nacional de Investigación y Atención en Quemados, Instituto Nacional de Rehabilitación, Mexico City 14389, Mexico; raulcarrilloesper@gmail.com

**Keywords:** environmental medicine, placental impairment, neurodevelopmental disorders, fetal brains, erythroblasts, preeclampsia, nanoparticles, NPs extracellular vesicles, petrochemical pollution, Villahermosa, Tabasco

## Abstract

Anthropogenic ultrafine particulate matter (UFPM) and industrial and natural nanoparticles (NPs) are ubiquitous. Normal term, preeclamptic, and postconceptional weeks(PCW) 8–15 human placentas and brains from polluted Mexican cities were analyzed by TEM and energy-dispersive X-ray spectroscopy. We documented NPs in maternal erythrocytes, early syncytiotrophoblast, Hofbauer cells, and fetal endothelium (ECs). Fetal ECs exhibited caveolar NP activity and widespread erythroblast contact. Brain ECs displayed micropodial extensions reaching luminal NP-loaded erythroblasts. Neurons and primitive glia displayed nuclear, organelle, and cytoplasmic NPs in both singles and conglomerates. Nanoscale Fe, Ti, and Al alloys, Hg, Cu, Ca, Sn, and Si were detected in placentas and fetal brains. Preeclamptic fetal blood NP vesicles are prospective neonate UFPM exposure biomarkers. NPs are reaching brain tissues at the early developmental PCW 8–15 stage, and NPs in maternal and fetal placental tissue compartments strongly suggests the placental barrier is not limiting the access of environmental NPs. Erythroblasts are the main early NP carriers to fetal tissues. The passage of UFPM/NPs from mothers to fetuses is documented and fingerprinting placental single particle composition could be useful for postnatal risk assessments. Fetal brain combustion and industrial NPs raise medical concerns about prenatal and postnatal health, including neurological and neurodegenerative lifelong consequences.

## 1. Introduction

Millions of people are exposed to environments with concentrations of fine particulate matter (PM_2.5_) above current USEPA standards, a well-known global risk factor of cardiovascular, respiratory, neurological and psychiatric morbidity and mortality [1,2,3,4]. Combustion, natural (soil, volcanic) ultrafine particulate matter (UFPM), and engineered nanoparticles (NPs) are key components of PM_2.5_ air pollution [5,6,7,8]. Their size (≤100 nm) and elemental composition, i.e., Fe, Hg, Ti, etc., makes UFPM and NPs highly reactive and cytotoxic, with a high capacity for damaging and crossing biological barriers [3,9,10,11,12,13,14]. Intrauterine life is a highly vulnerable developmental period for NP exposures, and maternal-fetal particle transfer leads to adverse health fetal and postnatal outcomes. Intrauterine toxicity and risk assessment of UFPM/NPs exposure during pregnancy is a subject of great interest, given that environmental and engineered NPs can cross the placenta [9]. NP (used here interchangeably with UFPM) size, shape, charge, surface composition, coating with biocompatible molecules, corona formation, and, certainly, stage of embryonic/fetus/placental maturation are key factors impacting free radical oxidative stress, inflammation, restricted placental growth, and the activation of placental toll-like receptors (TLRs), to name a few [9,10,11,12,13,14,15,16,17,18]. There is a deep concern regarding uterine environmental exposures, the developmental origins of disease, and the fetal programming model predicting lifelong consequences from early intrauterine and/or postnatal exposures to insults significant in length, cumulative doses, and properties favoring specific cell damage [19,20,21,22,23,24,25,26,27]. Experimentally, NPs cause fetal developmental toxicity, and the early stages of brain organogenesis are highly vulnerable to reactive oxygen species (ROS); ultrastructural alterations in mitochondria, endoplasmic reticulum (ER), and Golgi complexes; downregulation of neuronal glutamate transporters; and, ultimately, the impairment of cognition and alterations in animal behavior [23,24,25,26,27,28,29,30]. The early development of the radial microvasculature in the telencephalon cortical plate (CP) and the correct micro vessel formation are the basis for the later perivascular glia coverage formation and endothelial barrier maturation critical for setting up the brain–blood barrier (BBB) [31,32,33,34]. Thus, any damage to the primitive brain could be a crucial factor accounting for neurovascular unit (NVU) malfunction and/or dysfunctional glia and damaged neurons, resulting in perinatal pathologies and neurodegenerative disorders [35,36,37,38]. Metropolitan Mexico City (MMC) residents have been exposed to PM_2.5_ above the USEPA standards and nanoparticles [39,40], and a progressive development of Alzheimer’s disease starting in childhood has been described, along with brainstem hyperphosphorylated tau, α synuclein and TDP-43 pathology in young urbanites [41,42,43]. Critically, young MMC residents have the exogenous Fe-, Al-, and Ti-rich NPs in their brains associated with progressive neurovascular damage and quadruple aberrant protein pathology [41,42]. 

This pre-COVID study focuses specifically on the light and electron microscopy characterization of preeclamptic and normal-term placentas, and 12–15 week products and their placentas, from two polluted cities in Mexico: MMC and Villahermosa, Tabasco, whose populations have been continuously exposed to complex mixtures of air pollutants, including fine and ultrafine fractions of PM [41,42,43,44]. Given the passage of NPs from placenta to fetus in experimental animals and human stillbirths [9,45], we are also studying brains from postconceptional weeks PCW 12–15.

Our working hypothesis posits that in women chronically exposed to PM_2.5_ above the annual current US Environmental Protection Agency (USEPA) standard (12 µg/m^3^) and high NP concentrations, placental NPs could be taken as evidence for fetal–organ transfer [39,40,44]. We argue that the early-brain NP identification associated with structural organelle neural and blood vessel changes will have detrimental effects upon early neural cells and NVU formation and integrity, ultimately altering immature astroglial cells and morphogenesis and being a key factor for aberrant protein formation [31,32,33,34,35,46,47,48]. Highly neurotoxic combustion and engineered NPs are reaching fetal organs in development and are setting the bases for neurodevelopmental and neurodegenerative lifelong consequences.

## 2. Materials and Methods

### 2.1. Study Cities and Air Quality

Metropolitan Mexico City (MMC) and Villahermosa, Tabasco, were the selected cities because of high levels of metals in air pollution, with both locations having exposures to other PM_2.5_ sources (i.e., subway system and petrochemical activities). The MMC area covers ~7585 km^2^ and is located on an elevated basin 2240 m above sea level surrounded by mountain ridges. MMC has a population of ~21.8 million people. Emissions from ~6 million vehicles, over 50,000 industries, LP gas, industrial and household solvents, and vapors of oil-derived liquid fuels combined with high solar radiation and poor ventilation result in severe air pollution with a strong oxidizing capacity [49]. MMC residents have been exposed to high levels of primary fine and ultrafine particles, as well as secondary air pollutants including secondary organic aerosols and ozone concentrations, at levels above current United States National Air Ambient Quality Standards (NAAQS) all year round over the last two decades [39,40,50,51]. Commuting in any of the urban transport modes available in MMC is associated with high NP exposure [52,53]. Traveling in the Mexico City subway exposes residents to high PM_2.5_ concentrations between 34 and 93 μg m^−3^; NPs up to 50,300 ± 10,600 (# cm^−3^) at an average size of 38.5 ± 15.9 nm; and elevated concentrations of Fe, Cu, Ni, Cr, and Mn [52,53]. Villahermosa is the capital city of Tabasco and is located on an extended swampy plain at 20 m above sea level in southeast Mexico. Together with two neighboring municipalities, the city makes up the Metropolitan Area of Villahermosa (MAVH), covering around 2100 km^2^, with a population of ~860,000 inhabitants. Primary emissions from gasoline and heavy-duty diesel vehicles from urban and off-road transport add to the emissions of oil and gas production, compression and processing facilities, and biomass burning from the agricultural activities that surround the MAVH [54,55,56,57,58]. The MAVH region also receives air masses coming from off-shore oil and gas production platform flares in the Gulf of Mexico [54,56]. The MAVH’s atmosphere has a weakly reducing capacity versus the oxidizing nature of MMC’s atmosphere [54,55,57,58].

### 2.2. Preeclamptic, Normal Term, and Postconceptional Weeks (PCW) 12–15 Placentas and Products

This study was approved by two institutional review boards: the Committee of Ethics and Research at the Hospital General de México, Dr. Eduardo Liceaga, IRB: DI/17/112/03/037, and the Hospital Regional de Alta Especialidad, Dr. Gustavo A. Rovirosa Perez, Tabasco, IRB HR/073/2017, for the collection of mature and early PCW 12–15 placentas and products. This study included women ≥18 y and written consent was obtained from all participants with either term and preeclampsia pregnancies (placenta samples) or early placentas and products. Table 1 shows the demographic data on the 94 women participating in this study.

Placenta sections from normal term pregnancies and preeclampsia pregnancies *n* = 39 and 55 early pregnancy products (including 19 placentas) were examined. Experienced physicians (YDSCG, JCBG, AAPC) described the gross placental findings from all cases, rendering diagnoses per the Amsterdam guidelines [59]. Transmission electron microscopy (TEM) and energy dispersive X-ray spectrometry (EDX) studies were performed using three mm blocks from fetal organs and placentas. Samples were cut with ceramic knives and handled with plastic forceps free from metal contamination. High-angle annular dark field (HAADF) and scanning transmission mode (STEM) were used. A 300 kV FEG FEI TECNAI F30 transmission electron microscope (TEM), tuned for a 100 kV acceleration beam and an 11-spot size, were also utilized for examining the tissues supported on TEM nickel grids. We analyzed all samples blind to case and grid/tissue sections, and grid areas were randomly selected and methodically scanned. Electron microscopic analysis was conducted, as in previous works [42], to identify the NPs’ elemental compositions and the ultramicroscopic structures of maternal and fetal cells and organelles. The organelle NPs’ locations and their elemental metal and nonmetal contents were the focus of this study.

## 3. Results

### 3.1. Air Pollution

**Figure 1:**
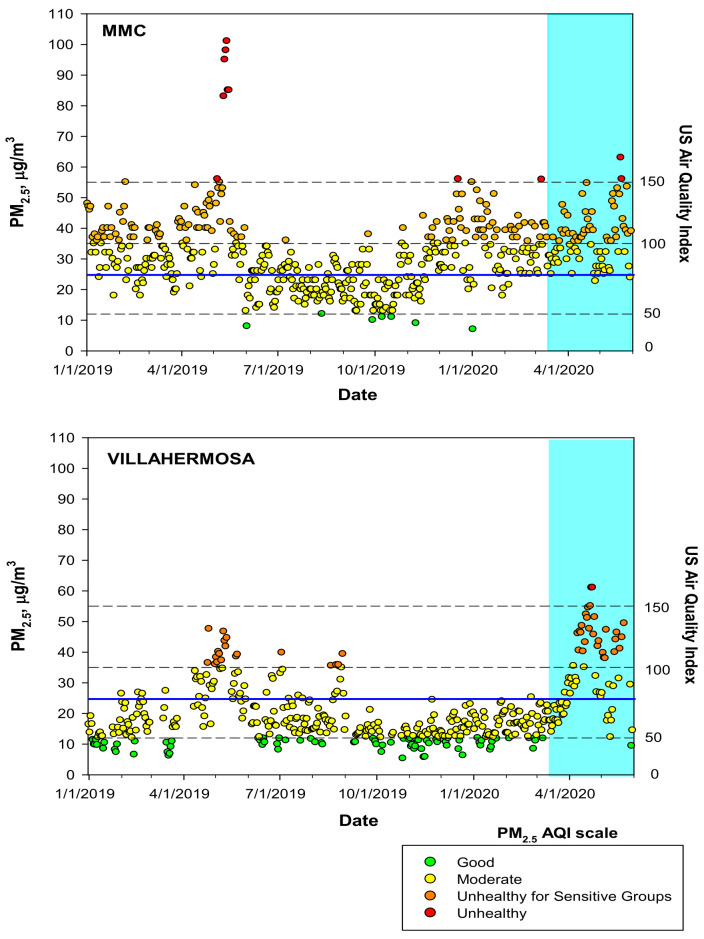
Time-series of maximum PM_2.5_ 24-h averages at MMC and estimated daily 24-h averages at MAVH January 2019 to May 2020—including the 11 m study period—classified according to the US EPA AQI index. The blue continuous line depicts the 24-h average WHO guideline. The blue shade area represents the beginning of the COVID-19 official lockdown in Mexico. Air quality data were available from the Sistema de Monitoreo Atmosférico del Gobierno de la Ciudad de México (http://www.aire.cdmx.gob.mx/default.php, accessed on 29 December 2021 and Sistema Nacional de Información de la Calidad del Aire. Instituto Nacional de Ecología y Cambio Climático (INECC), México (http://sinaica.inecc.gob.mx/data.php, accessed on 29 December 2021).

MMC and MAVH residents have been chronically exposed to PM_2.5_ with concentrations above the current USEPA annual standard. Figure 1 shows the time-series for the maxima PM_2.5_ 24-h average registered by the official monitoring network in MMC and the daily 24-h averages for the MAVH monitoring stations according to the US EPA AQI-Index for the period January 2019 to May 2020, which includes the 11-month study period in 2019.

### 3.2. Placental Light Microscopy and Transmission Electron Microscopy (TEM)

**Figure 2:**
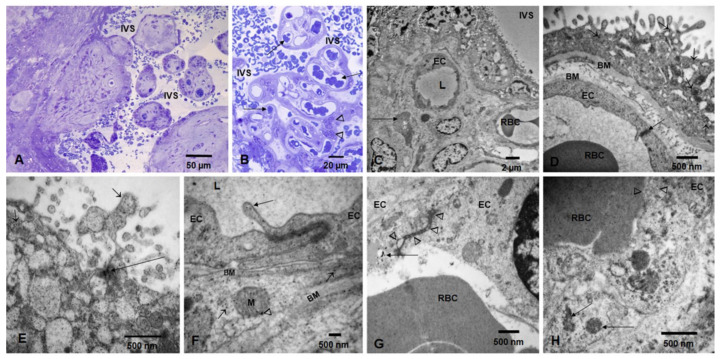
Toluidine blue 1 um thick sections and TEM in term placentas. Toluidine blue 1 um thick sections showing term villi surrounded by intervillous space (IVS) occupied by maternal RBC (**A**). Thin syncytiotrophoblast and numerous blood vessels (long arrows) occupied by fetal RBC and Hofbauer cells (arrowheads) are seen in (**B**). Electron micrograph showing the IVS, syncytiotrophoblast, fetal blood vessels, endothelial cells (EC) with luminal (L) RBC, and a Hofbauer cell (long arrow) (**C**,**D**). A close-up of a villous shows the syncytiotrophoblast layer resting on a basement membrane (BM upper) with numerous NPs (short arrows). A fetal endothelial cell (EC) contains a fetal RBC, and an intact EC tight junction is seen (long arrow) (**E**). Close up of syncytiotrophoblast with numerous free NPs (short arrows) and clusters of NPs (long arrows). (**F**). Fetal blood vessel with two ECs and a tight junction (TJ). Note the prolongation into the lumen (L) of the endothelial cell containing NPs (long arrow). The EC is resting on a basement membrane (BM upper). A pericyte surrounds the ECs and contains cytoplasmic free NPs (short arrows) and NPs in mitochondria (arrowhead). The pericyte basement membrane is visible (BM lower). (**G**). Fetal ECs with an abnormal TJ (arrowheads), occupied by NPs and an adjacent EC lysosome, contains numerous NPs (long arrow). The fetal RBC is seen in vessel lumen. (**H**). One fetal luminal RBC in close contact with the EC shows an area of intense caveolar activity (arrowheads) and NPs lining the contact surface. ECs contain numerous lysosomes with NPs (long arrows).

Term placentas showed villi corresponding to third trimester pregnancies surrounded by intervillous space (IVS) occupied by maternal red blood cells (RBC) (Figure 2A), as well as thin syncytiotrophoblast and numerous blood vessels occupied by fetal RBC (Figure 2B). Electron micrographs show the IVS, syncytiotrophoblast, fetal blood vessels, endothelial cells (ECs), and Hofbauer cells (Figure 2C,D). Syncytiotrophoblast with numerous free NPs, as well as clusters of NPs and fetal blood vessels with ECs, can be seen in Figure 2E,F. Fetal ECs with abnormal tight junctions (TJ) occupied by NPs and fetal luminal RBC in close contact with the EC can be seen in Figure 2G,H.

**Figure 3:**
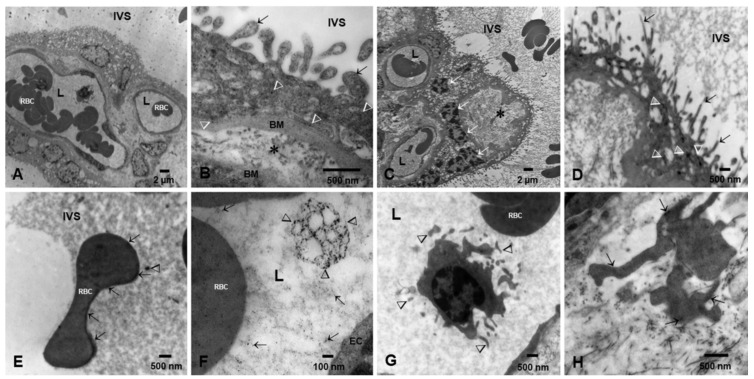
Preeclamptic placentas TEM. (**A**). Villous cores with vacuolated syncytiotrophoblast and few fetal blood vessels lumen (L) containing fetal RBC and white blood cells (WBC). The maternal IVS can be seen. (**B**). A close-up of a villous section with numerous NPs (arrow heads) throughout the layer and also in the prolongations into the IVS (short arrows). The cytotrophoblast basement membrane is irregularly thick (BM), also contain NPs, and is separated from the EC basement membrane by abundant collagen tissue (*). (**C**,**D**) are examples of severe preeclamptic cases with thin syncytiotrophoblast and numerous clusters of pyknotic nuclei (white arrows) in the midst of vacuolated areas (*). Fetal blood vessels are few, ECs are flat and thin, and the lumen (L) is occupied by RBC. (**D**) shows a close-up of a syncytiotrophoblast with thin extensions (short arrows) into the IVS and numerous NPs with elongated shapes (arrow heads). (**E**). This is a maternal RBC in the IVS showing abundant NPs lining the surface of the cell. Free NPs are also observed (arrowhead). (**F**). Close-up of a fetal blood vessel lumen (L) and EC showing a ~600 nm irregular exosome decorated with numerous NPs (arrowheads); also notice the free lumen NPs (short arrows). (**G**). In the same fetal blood vessel as (**A**), a monocytic WBC is seen at high power with its branched projections (short arrows). In the midst of the collagenous fibers, fibroblastic cell processes display numerous NPs (short arrows) (**H**).

In contrast, preeclamptic low-weight placentas showed vacuolated SCT, villous cores with few fetal vessels, and numerous NPs in the SCT and basement membranes (Figure 3A–H). Maternal RBC in the intervillous space (IVS) contained large numbers of NPs (Figure 3E), while fetal RBCs in fetal vessels showed numerous NPs and extracellular vesicles (EVs) decorated with NPs (Figure 3F).

**Figure 4:**
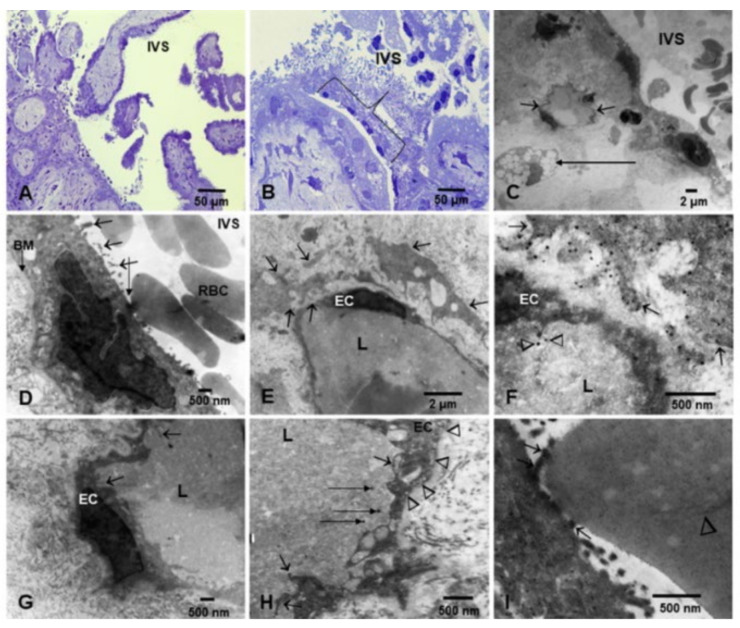
Placentas postconceptional weeks (PCW) 8–15, toluidine blue sections, and TEM. Toluidine blue sections showed mesenchymal villi with loose stroma surrounded by cytotrophoblast and syncytiotrophoblast, Hofbauer cells, mesenchymal, and fibroblast like cells (**A**). A section of syncytiotrophoblast is shown in (**B**) ({). Low power TEM (**C**) shows villi with loose stroma rich in mesenchymal cells, a fetal blood vessel (short arrows), and a Hofbauer cell (long arrow). Placental villi float into the maternal blood in the intervillous space (IVS). (**D**). Close-up of the syncytiotrophoblast (SCT) in close contact with maternal RBC in the IVS. There is a close contact between the SCT and the maternal RBC, and a cluster of NPs in the area (long arrow). The SCT prolongations into the IVS are marked by short arrows. (**E**). A fetal blood vessel is surrounded by two fibroblastic-like cells with numerous cytoplasmic prolongations surrounding the vessel (short arrows). (**F**). One fibroblastic-like cell around the fetal blood vessel is seen with numerous NPs (short arrows). An endothelial fetal cell (EC) shows caveolar activity and luminal (L) free NPs are marked with arrowheads. (**G**). A fetal EC shows intense filopodia activity—cytoplasmic prolongations reaching towards the lumen (short arrows). (**H**). Fetal ECs with intense caveolar and filopodia activity. The caveolar activity is intense both at the luminal (short arrows) and subluminal surfaces (arrowheads). Free luminal (L) NPs are also present (long horizontal arrows). (**I**). Close contact between an erythroblast with fragmented nuclear remnant (arrowhead) and the fetal EC with filopodia loaded with NPs (short arrows).

Placentas at PCW 12–15 can be seen in Figure 4. The toluidine blue sections showed mesenchymal villi with loose stroma surrounded by cytotrophoblast and syncytiotrophoblast (Figure 4A,B). Electron micrographs (Figure 4C–I) showed villi with loose stroma rich in mesenchymal and Hofbauer cells. Maternal RBC containing NPs were seen in close contact with early SCT (Figure 4D). Fetal erythroblasts showed close contact with endothelial fetal blood vessels (Figure 4I).

### 3.3. Fetal Brains Light Microscopy and TEM

**Figure 5:**
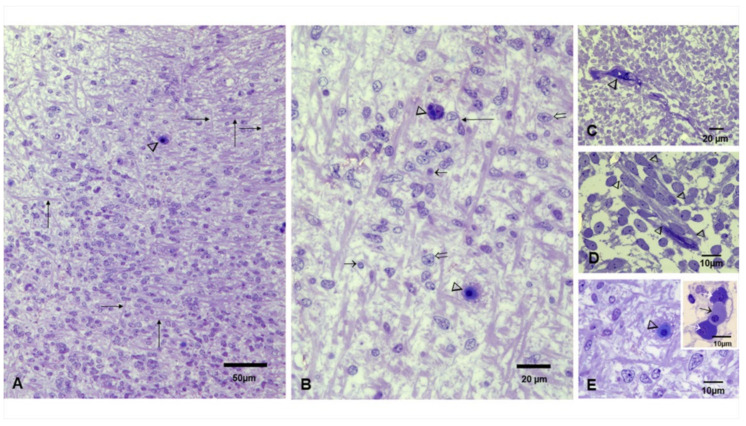
Brain toluidine blue sections, postconceptional weeks (PCW) 12–15. (**A**). Frontal region with a dominant radial organization, fibers in horizontal and vertical orientation (long arrows), and small blood vessels occupied by erythroblasts can be identified (arrowhead). (**B**). Close-up with fibers oriented in horizontal and vertical directions along cells with diverse nuclei shapes (short and open arrows) interspersed by cells with long processes (long arrow) and blood vessels occupied by erythroblasts (arrowheads). (**C**). Primitive glial cells can be identified along with small vessels (arrowhead). (**D**). Slender cells with long processes and vesicular nuclei streaming (arrowheads) in the primitive cortex. (**E**). Neuronal bodies and numerous capillaries with luminal erythroblasts areidentified. Insert shows three erythroblasts in different stages of development; the orthochromatic cell is marked with a short black arrow between two basophilic cells marked with white arrows.

The representative toluidine blue sections in Figure 5 show the early fetal phase post-conceptional weeks (PCW) 8–12.5 and the early fetal period 13–15 PCW [60]. Numerous small blood vessels were seen throughout the cortical plate with luminal erythroblasts (Figure 5A–E). Primitive glial cells were identified along neuronal bodies and capillaries with luminal erythroblasts (Figure 5B,D,E and insert).

**Figure 6:**
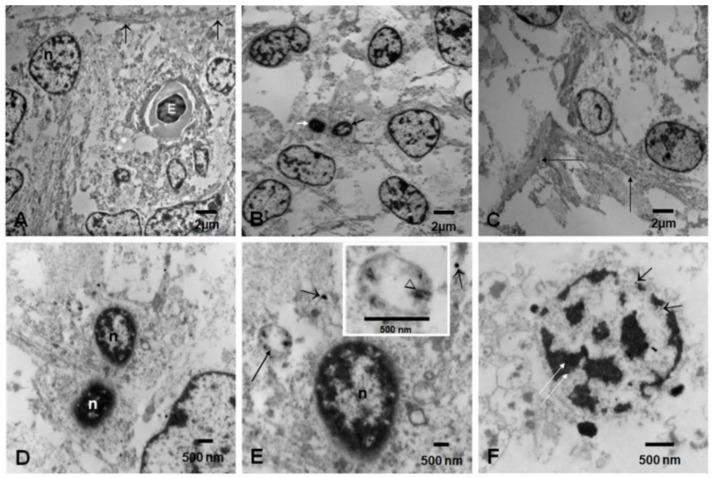
Electron microscopy of brain cortical cells. (**A**). Electron microscopy of cortical areas showed capillaries occupied by erythroblastic cells (E) with large nuclei, surrounded by primitive neural cells and horizontal fibers (arrows). (**B**). A number of neural primitive cells show large lobulated, medium size, and small nuclei (small black and white arrows). (**C**). Bundles of fibers (arrows) can be seen crossing the primitive neuropil. (**D**). Two cells with small distinct nuclei- the pyknotic one marked with a white (n) are conspicuous because of their cytoplasmic features seen in (**E**). (**E**). The same cell as in (**D**) contains mitochondria with numerous nanoparticles (long arrow) and free cytoplasmic NPs (short arrows). Insert: the particulate nanomaterial exhibits rod and spindle shapes (arrowhead). (**F**). A primitive neuronal nucleus with NPs free (short black arrows) in the nuclear matrix and associated with heterochromatin (long white arrows).

Electron microscopy of the cortical structures showed capillaries occupied by erythroblasts with large nuclei surrounded by primitive neural cells (Figure 6A–C). Cells with darker and smaller nuclei (Figure 6D–F) exhibited mitochondria with numerous NPs with acicular and spherical shapes. NPs were also seen free in the cytoplasm and inside the nucleus (Figure 6F).

**Figure 7:**
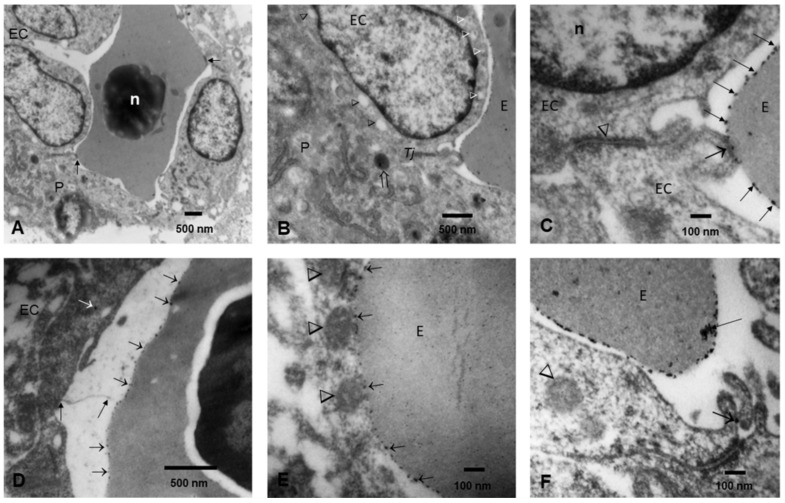
Brain blood vessels postconceptional weeks (PCW) 12–15 (**A**). Brain fetal vessel is occupied by one erythroblast with a dark, disintegrating nucleus (n). Close contact between the erythroblast and the endothelial cell (arrows). A pericyte is identified as P. (**B**). Endothelial cells (EC) display luminal (white arrowheads) and subluminal (black arrowheads) intense caveolae activity, with some caveolae containing NPs. A tight endothelial junction is marked Tj, and the erythroblast is marked E. A pericyte contains lysosomes (open white arrow) containing NPs. (**C**). A close-up of the EC tight junction (arrowhead) decorated with NPs adjacent to finger-like projections, with numerous caveolae attached to the surface of an erythroblast displaying numerous surface NPs (arrows). (**D**). Slender EC filopodia (two long black arrows) reaching towards the erythroblast displaying surface NPs (short arrows). The EC displays one free cytoplasmic NP (white arrow). (**E**). An erythroblast with a large surface studded with NPs (short arrows) in close contact with EC lysosomes (arrowheads). (**F**). Erythroblast with numerous surface NPs, with some forming conglomerates (long arrow). Endothelial filipodia display a single NP in caveola (short arrow). A lysosomal structure with NPs is identified in the EC cytoplasm (arrowhead).

Numerous brain fetal capillaries were occupied by erythroblasts, and the endothelium displayed free NPs in the cytoplasm and inside lysosomes, along with intense luminal and subluminal caveolar activity (Figure 7A–F). Membrane-coated caveolae contained isolated NPs on the abluminal and the luminal EC sides, and slender EC finger-like projections reached towards the erythroblasts’ surface, studded with NPs. Endothelial tight-junction (TJ) complexes, localized along the lateral membrane, were decorated with NPs. Pericytes were identified in small vessels showing lysosomal structures with NPs and abundant rough endoplasmic reticulum.

### 3.4. Energy Dispersive X-ray Spectrometry

**Figure 8:**
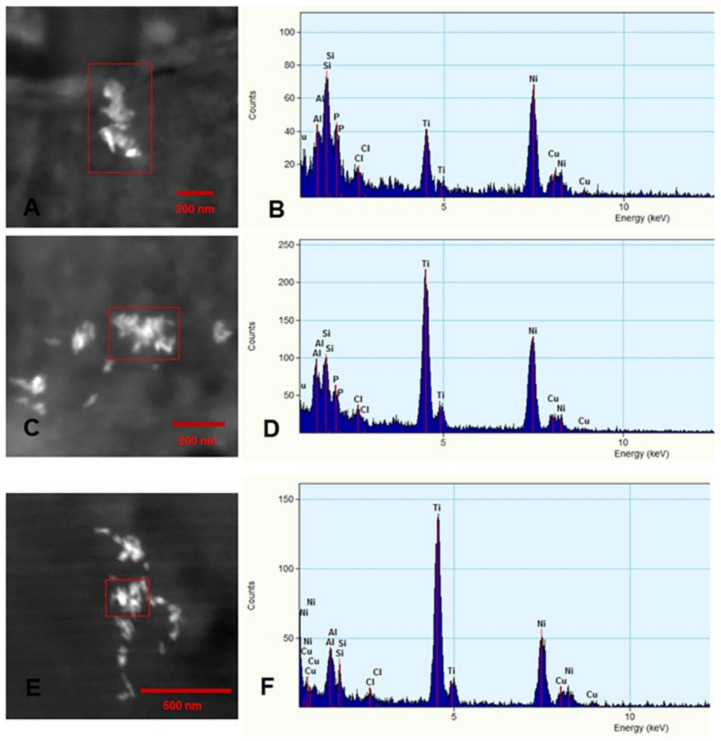
Representative nanoparticles with EDX elemental maps from term and early placenta and brain PCW 12 samples. Panels (**A**,**B**): term placenta shows an agglomerate of NPs containing titanium (ranging from 10 to 100 nm) co-occurring with silicon and aluminum. Panels (**C**,**D**): early placenta at PCW 12 with an agglomerate of titanium, silicon and aluminum. Panels (**E**,**F**): brain samples at PCW 12 with titanium, aluminum, and silicon. Ni grids were used.

EDX elemental mapping was used to identify the elemental distribution in all analyzed particles, and Figure 8 shows the representative profiles of metal and nonmetal NPs. Titanium-containing particles were frequently detected in the placenta (Figure 8A–D) and brain tissues (Figure 8E,F). Mixtures of Si, Al, and Ti nanorods were common.

**Figure 9:**
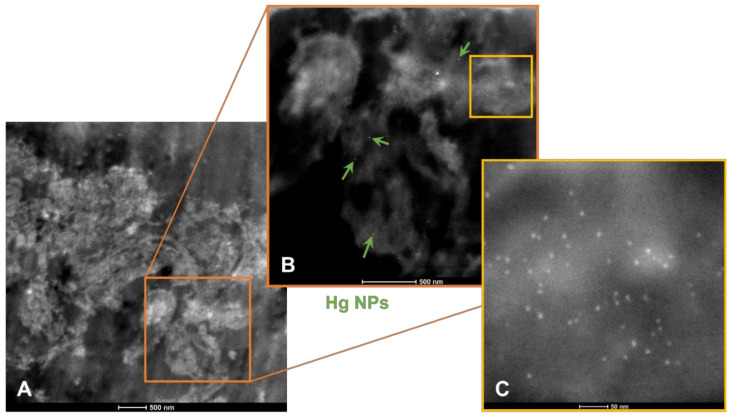
Composite picture of Hg and Fe nanoparticles in telencephalic PCW 12 brain tissues. In panel (**A**), low power view of the sampled brain tissues; in panel (**B**), the brightest nanoparticles are composed of Hg (7–12 nm in range) and are marked with green arrows, and all other NPs are Fe. Panel (**C**) contains Fe NPs mostly in the 4–6 nm range.

Mercury (Hg) and Fe containing particles were detected in brain samples (Figure 9).

## 4. Discussion

Combustion and engineered environmental nanoparticles are reaching brain tissues at early human developmental PCW 8–15 stage, and documentation of NPs in both the maternal and fetal placental compartments at early, preeclamptic, and normal term placentas strongly suggests the placental barrier is not limiting the access of highly toxic NPs.

NPs are reaching the brain at critical stages: PCW 8–12.5, characterized by proliferation, migration, and molecular specification; followed at PCW 13–15 by cell aggregation, proliferation, migration, neuronal and dendritic differentiation, and axonal growth, according to Kostović et al. [60], a highly recommended and important work. Data of increasing blood flow to the intervillous space as early as 6 weeks of gestation makes the possibility of NP erythrocyte maternal transport to early developmental embryonic stages plausible [61].

Erythroblasts are identified in this work as the main early NP carriers to fetal tissues. Erythroblasts are fundamental in the transition from rapidly growing embryo to the fetus and colonize the bone marrow at 10.5 weeks [62]. Moreover, since active circulation starts approximately at day 29, erythroid cells are likely involved in NPs’ transportation from early stages.

Experimentally, the internalization of nanosized materials is a complex process that involves direct membrane penetration with NPs free in the cytosol and endocytotic uptake via biomembrane-coated vesicles, requiring NP sizes in the range of 10–100 nm [63,64,65]. The presence of highly reactive Fe-rich and Hg NPs ≤ 10 nm inside brain cells brings up the transportation pathway discussed by Panja and Jana [65]: arginine-terminated Au ≤10 nm enters via energy-independent direct membrane penetration and, as the size increases, there is a switch to energy-dependent endocytotic uptake. A critical issue at stake involves the fetal brain endothelial and pericyte tight junctions (TJs) and lysosomal NPs’ accumulation at PCW 8–15 weeks, potentially impacting the brain–blood barrier (BBB) formation, its regulation, and permeability [66,67,68]. The BBB is functional as early as 8 weeks of gestation [67,69]; thus, the documentation of brain parenchyma and blood vessels’ NPs at PCW 8–15 is of deep concern regarding the potential damage to the incipient BBB. 

A major finding in this work was the early pregnancy NPs’ placental profile characterized by multiple nanoscale deposits of environmentally-sourced Fe, Ti, Cu, Ca, Sn, Al, and Si. Isolated Si NPs were common and seen in combination with Al and Ca. In this work, Fe and Ti were commonly present in brain NPs, adding to the high production of free radicals and the documented effective promotion of fibrillation of key neural proteins as alpha synuclein [70,71,72,73], an extra concern for airborne magnetic mixtures of spherical and elongated NPs and their response to magnetic forces, i.e., heat production under high-frequency alternating magnetic or near-infrared optical fields and increased oxidative stress [74,75,76,77]. Vereda et al. [74] discussed the magnetization and friction coefficient anisotropies in elongated Fe NPs versus spherical ones, a major concern in our early placental samples. Ovejero and colleagues [76] discussed that the mixing of NPs modulates magnetic responses and their thermal evolution under alternating magnetic fields. Abu-Bakr and Zubarev [75] commented on heat production by clusters of single-domain ferromagnetic particles and two scenarios of strong and weak magnetic anisotropy; i.e., in strong anisotropy, the particle clusterization weakens the thermal effect, whereas weak anisotropy enhances it. Sola-Leyva and colleagues [77] showed that a low concentration of magnetic NPs under a low intensity alternating magnetic field (AMF) increased the production of intracellular ROS. Their results demonstrated that intracellular ROS production increases up to ∼90% following the exposure of AMF to HepG2 cells containing biomimetic magnetic NPs and result in a 40% loss of cell viability without a significant rise in temperature.

Key NP researchers’ work [74,75,76,77,78,79] fills a substantial knowledge gap potentially applicable to fetuses exposed to NPs, pointing to the early and significant involvement of the nanosized combustion, friction, and electronic waste associated with organelle structural changes, oxidative stress, and magnetic damage.

Conspicuously, silica was abundant in early placentas, and its association with early villi is worth discussing. The in vivo work of Li J et al. [80], designed to examine the uterine accumulation of SiNP using FITC coupled onto SiNPs in pregnant mice, showed SiNP penetrating the trophoblast membrane, leading to apoptosis, the suppression of cell proliferation, tube formation, and invasion in a dose-dependent manner. SiNP also induced uterine inflammation in vivo [80]. Additionally, genotoxicity both in vivo and in vitro [81] is described with SiNPs < 21 nm and, thus, seemingly harmless Si nanostructures associated to Al and Ca in the typical airborne pollution mixtures compound the synergistic genotoxic and oxidant early placental damage.

The presence of Ti nanorods in all placental samples and in the fetal brain is a strong warning that nano-engineered NPs (i.e., food, waste electrical and electronic equipment (WEEE)) are also involved in placental and neural damage [82,83,84,85].

The detrimental NP effects upon brain morphogenesis and microvasculature development are of deep concern. The in vitro work of Coccini and colleagues [47] is relevant: NP uptake resulted in a reduction in neuronal differentiation with a downregulation of β-tubulin III, microtubule-associated protein 2, enolase, and nestin. A dose-related effect was recorded, and the gene downregulation persisted for up to 8 days without cell morphology alterations.

Remarkably, the identification in preeclamptic placentas’ fetal vasculature of extracellular vesicles (EV) loaded with NPs could result in a negative impact at the fetal-maternal interface and act as a bio-signaling paradigm [86,87,88,89,90]. The EV capacity to impact neurodevelopmental pathologies is an important issue, particularly because we detected them in preeclamptic placentas from petrochemical pollution-exposed women. This information is relevant to the strong association between air pollution and preeclampsia, prematurity, fetal growth restriction, uterine inflammation, and abnormal placenta vascularization [88,91,92,93,94]. Moreover, EVs have been associated with preeclampsia pathophysiology, CNS developmental disorders, and regarded as potential early biomarkers for adverse NP exposure [88,89,94].

The placenta is at the core of the interface between mother and fetus [22,27,95].

The placental barrier is not an unfailing barrier, and the issue of neurodevelopmental and neurodegenerative trajectories with serious short- and long-term impacts ought to be entertained. The vulnerability issue was reviewed by Back [96] for preterm brain and its great susceptibility to cerebral white matter injury disrupting the normal progression of developmental myelination. Tracts form along the path traced by the “pioneer axons”, which are guided by various molecular cues [97]. This is an example of a highly precise phenomenon, during which any disturbance can have serious consequences in postnatal life.

Urcini et al. [27] have detected an interaction between genomic risk scores for schizophrenia (GRSs) and early-life complications (ELCs), based on placental gene-expression loci (PlacGRSs). Strikingly, the relationship of PlacGRSs with brain volume persists in adults—mostly males—with schizophrenia, defining a potentially preventable neurodevelopmental path of risk applicable to schizophrenia but open to a number of neurodevelopmental and neurodegenerative diseases.

We are very aware that healthy pregnancies and products depend on proper maternal-fetal interactions, starting with a healthy mother, residing in a clean environment, proper fertilization, embryo implantation, unremarkable placental development, normal vascular function both in the mother and the product, balanced nutrition, and no exposures to toxicants during the entire pregnancy [13,98,99].

There are limitations and advantages to the study. We only included placental and fetal tissues from highly exposed mothers, essentially because the highly specialized obstetric hospitals in Mexico are located in large polluted cities. Unfortunately, we had no support to purchase Au grids, nor had access to state of the art HAADF and STEM equipment. A major advantage of our study was the multidisciplinary collaborators and the efforts made to coordinate and exchange viewpoints regarding the importance of placental changes and fetal NPs, which are key to understanding the impact of our findings in future studies.

## 5. Conclusions

Placental, embryonic, and fetal toxicity are at the core of the adverse outcomes of nanoparticles. The vulnerability of the brain is key, essentially because there is no question that a number of chemicals, and certainly NPs, can interfere with the highly precise neurodevelopmental processes taking place in intrauterine life [9,10,11,12,13,14,15,16,17,18,24,25,26,27,45,46,47,48,91,96]. Of critical importance is the relationship between intrauterine toxic exposures and risk for major neurological, psychiatric, and cardiovascular diseases, a subject discussed for a number of years since the pioneering work of Barker [19] and 21 century researchers discussing fetal and perinatal programming and neuropsychiatric, metabolic, and cardiovascular diseases [20,21,22,23,24,25,26,27].

The problem is complex, the questions are countless, and the answers restricted: (1) we know very little about NPs’ direct and indirect effects on human placentas and fetuses; (2) the placenta does not provide a significant physical NP barrier for fetal protection starting in early pregnancy; (3) the detection of Hg NPs in the telencephalic brain is extraordinarily important and concerning for all researchers. Mercury is ubiquitous in volcanic active regions, and anthropogenic activities release large amounts of Hg into the environment [100]. The chemical speciation of Hg determines its mobility and toxicity, and the fetal brain is particularly vulnerable, with accumulated Hg concentrations 5–7 times higher than in maternal blood [101,102,103,104,105,106]; (4) NPs have extraordinary variations in terms of sources, chemical composition, shape, size, valence, corona formation, etc., which means that we are in a very difficult position to define and to study their direct effect upon placentas and fetuses across pregnancies, diverse environments, and NP sources; (5) genetic, nutritional, and metabolic maternal factors play a role in the fetal response to neurotoxicants.

Our concern as obstetricians and gynecologists should not end with learning about preeclampsia, preterm birth, fetal growth restriction, or gestational diabetes mellitus, but should also include collecting and sharing prenatal information to look for postnatal short and late events, including the risk of the major neurodegenerative diseases responsible for significant morbidity and mortality in our populations.

The need for multidisciplinary, noncoercive, cooperative research groups is very clear. Intrauterine life is a highly vulnerable period for NP exposures. Our findings can immediately inform preventive measures, clinical care, and deployment strategies to maximize benefits for pregnant women and their products across the world.

## Figures and Tables

**Table 1:** Demographic data on the 94 women participating in this study.

Tissues	Residency	Women Age	Pregnancy Weeks
Term Normal Placentas *n* = 7	MMC	27.7 ± 7.29 y	39.12 ± 2.44
Preeclampsia placentas *n* = 7	MMC	25.42 ± 6.5 y	37.02 ± 2.47
Term Normal Placentas *n* = 12	Villahermosa	26.91 ± 4.1 y	38.25 ± 1.48
Preeclampsia placentas *n* = 13	Villahermosa	22.07 ± 6.96 y	36.32 ± 3.14
Early placentas *n* = 19 *	Villahermosa	27.21 ± 6.50 y	12–15 weeks
12–15 week products *n* = 55	Villahermosa	26.01 ± 6.27 y	12–15 weeks

* The 19 cases with early placenta samples belong to the 55 group.

## Data Availability

Data supporting reported results are included in this work.
